# Gene correction and overexpression of *TNNI3* improve impaired relaxation in engineered heart tissue model of pediatric restrictive cardiomyopathy

**DOI:** 10.1111/dgd.12909

**Published:** 2024-01-09

**Authors:** Moyu Hasegawa, Kenji Miki, Takuji Kawamura, Ikue Takei Sasozaki, Yuki Higashiyama, Masaru Tsuchida, Kunio Kashino, Masaki Taira, Emiko Ito, Maki Takeda, Hidekazu Ishida, Shuichiro Higo, Yasushi Sakata, Shigeru Miyagawa

**Affiliations:** ^1^ Department of Cardiovascular Surgery Osaka University Graduate School of Medicine Osaka Japan; ^2^ Premium Research Institute for Human Metaverse Medicine Osaka University Osaka Japan; ^3^ NTT Communication Science Laboratories Media Information Research Department Kanagawa Japan; ^4^ Department of Pediatrics Osaka University Graduate School of Medicine Osaka Japan; ^5^ Department of Medical Therapeutics for Heart Failure Osaka University Graduate School of Medicine Osaka Japan; ^6^ Department of Cardiovascular Medicine Osaka University Graduate School of Medicine Osaka Japan

**Keywords:** engineered heart tissue, induced pluripotent stem cells, relaxation impairment, restrictive cardiomyopathy, TNNI3

## Abstract

Research on cardiomyopathy models using engineered heart tissue (EHT) created from disease‐specific induced pluripotent stem cells (iPSCs) is advancing rapidly. However, the study of restrictive cardiomyopathy (RCM), a rare and intractable cardiomyopathy, remains at the experimental stage because there is currently no established method to replicate the hallmark phenotype of RCM, particularly diastolic dysfunction, in vitro. In this study, we generated iPSCs from a patient with early childhood‐onset RCM harboring the *TNNI3* R170W mutation (R170W‐iPSCs). The properties of R170W‐iPSC‐derived cardiomyocytes (CMs) and EHTs were evaluated and compared with an isogenic iPSC line in which the mutation was corrected. Our results indicated altered calcium kinetics in R170W‐iPSC‐CMs, including prolonged tau, and an increased ratio of relaxation force to contractile force in R170W‐EHTs. These properties were reversed in the isogenic line, suggesting that our model recapitulates impaired relaxation of RCM, i.e., diastolic dysfunction in clinical practice. Furthermore, overexpression of wild‐type *TNNI3* in R170W‐iPSC‐CMs and ‐EHTs effectively rescued impaired relaxation. These results highlight the potential efficacy of EHT, a modality that can accurately recapitulate diastolic dysfunction in vitro, to elucidate the pathophysiology of RCM, as well as the possible benefits of gene therapies for patients with RCM.

## Graphical abstract

In this study, we demonstrated that R170W induced pluripotent stem cell‐derived cardiomyocytes (R170W‐iPSC‐CMs) and engineered heart tissue (EHTs) exhibit prolonged relaxation time and relaxation impairment and established an in vitro evaluation model. Furthermore, gene correction and overexpression of *TNNI3* improved relaxation impairment in iPSC‐CMs and ‐EHTs. The EHT modality can precisely recapitulate the impaired relaxation of restrictive cardiomyopathy (RCM) in vitro and therefore may serve as a gateway to insight into RCM pathogenesis and the development of novel therapeutic strategies.

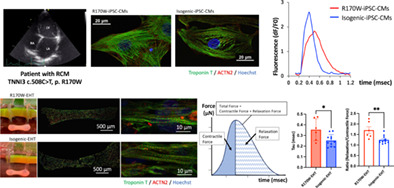

## 1 INTRODUCTION

Restrictive cardiomyopathy (RCM) is a rare form of primary cardiomyopathy characterized by ventricular diastolic dysfunction with preserved contraction (Maron et al., 2006). Its incidence rate is low, accounting for only 4.5% of pediatric cardiomyopathies (Lee et al., 2017), but it has a poor prognosis. The 5‐year transplant‐free survival rate in patients with RCM is 34%–38% (Anderson et al., 2018; Russo & Webber, 2005; Webber et al., 2012), which is lower than that in patients with other major pediatric cardiomyopathies; for example, it is 70% for dilated cardiomyopathy (DCM) (den Boer et al., 2015; Towbin et al., 2006) and 95% for hypertrophic cardiomyopathy (HCM) (Conway et al., 2023; Nguyen et al., 2023). Currently, the only curative treatment for RCM is heart transplantation, and considering the shortage of pediatric heart donors in Japan, alternative therapies are needed.

Human induced pluripotent stem cell (iPSC) technology is a powerful tool for studies of patient‐specific pathophysiology in the rare disease area (Bellin et al., 2012; Feric & Radisic, 2016; Matsa et al., 2014; Yang et al., 2014; Yazawa et al., 2011). Nonetheless, there are only a few published models of RCM using iPSCs, in contrast to the numerous reports on DCM and HCM. One reason for the limited number of RCM studies using iPSCs is the challenge of reproducing and assessing diastolic dysfunction, which is characteristic of RCM, in vitro. The degree of accuracy of in vitro representations mimicking diastolic dysfunction observed in patients with RCM without systolic dysfunction at the cardiomyocyte level is currently unclear. Relaxation time is frequently used as a metric for studying contraction–relaxation kinetics in cardiomyocytes or myocardial tissues (Jaferzadeh et al., 2023; Ronaldson‐Bouchard et al., 2018; Wang et al., 2023b). Furthermore, a recent study on engineered cardiac tissue (ECT) using iPSCs derived from a patient with RCM reported increased relaxation time in the ECT of this patient (Wang et al., 2023a). This study demonstrated for the first time that the contractile kinetics of mature ECT may represent the clinical phenotype of diastolic dysfunction in RCM.

The pathogenesis of cardiomyopathy is multifaceted, and several factors contributing to RCM have been examined. As with other cardiomyopathies, genetic mutations in sarcomere proteins have been implicated in RCM, with a genetic variant found in 25%–50% of patients with RCM (Kostareva et al., 2016; Lee et al., 2017). Genetic analysis of patients with RCM has identified potentially pathological variants in numerous genes, including *TNNI3*, *TNNT2*, *MYL2*, *FLNC*, and *MYH7* (Kostareva et al., 2016; Mogensen et al., 2003; Rindler et al., 2017). Among these, *TNNI3* mutations are the most common in RCM and result in a poor prognosis (Ishida et al., 2023). However, only few studies have used iPSC‐based pathological models of cardiomyopathy with *TNNI3* mutations. A previously reported ECT model with diastolic dysfunction was derived from an RCM patient with an *FLNC* mutation (Wang et al., 2023a).

TNNI3 is one of the three isoforms of Troponin I and is exclusively expressed in the heart. During the developmental transition from the fetal to neonatal and postnatal periods, TNNI3 is upregulated and replaces TNNI1 as the sole isoform in the adult heart (Sasse & Kyprianou, 1993; Sheng & Jin, 2016). This TNNI isoform switch from TNNI1 to TNNI3 serves as an important quantitative marker of the maturation status of iPSC‐derived cardiomyocytes (iPSC‐CMs) (Bedada et al., 2014). iPSC‐CMs exhibit an immature cardiac phenotype similar to fetal cardiomyocytes, but as they mature, various functional maturations have been observed along with increased TNNI3 expression (Fujiwara et al., 2023; Miki et al., 2021). Cardiac maturation is a major obstacle in experimental disease models using iPSC‐CMs, which may account for the lack of in vitro studies of diseases caused by TNNI3 abnormalities.

Herein, we generated an iPSC line from a child with early‐onset severe RCM carrying a mutated *TNNI3* gene and its isogenic line, and we analyzed calcium kinetics using the iPSC‐CMs. Furthermore, we demonstrated that mature engineered heart tissue (EHT) is an advantageous model of the RCM phenotype of impaired relaxation. We have also proven that overexpression of wild‐type *TNNI3* ameliorates the RCM phenotype with the *TNNI3* R170W mutation, suggesting a potential therapeutic target for RCM patients with *TNNI3* mutations.

## 2 MATERIALS AND METHODS

### 2.1 Human samples

Clinical data and samples were collected from the patient with the approval of the Ethics Committee of Osaka University Hospital. Written informed consent for the use of samples and genomic analysis was obtained from the patient before the harvest of blood mononuclear cells. This investigation was conducted in accordance with the Ethical Guidelines for Medical and Health Research Involving Human Subjects in Japan and the principles outlined in the Declaration of Helsinki.

### 2.2 Generation of patient‐derived iPSCs and isogenic iPSCs

Patient‐derived iPSCs were generated from the peripheral blood mononuclear cells (PBMCs) of a pediatric RCM patient harboring a heterozygous *TNNI3* mutation (c.508C>T, p.R170W). Briefly, PBMCs were isolated from whole peripheral blood using Histopaque (Sigma‐Aldrich). Reprogramming was performed by Sendai virus vectors (CytoTune‐iPS 2.0 Sendai Reprogramming Kit; Thermo Fisher Scientific). After 24 h of infection, PBMCs were seeded onto a laminin‐coated plate (iMatrix‐511, MATRIXOME). iPSCs were cultured under feeder‐free conditions using StemFit AK02N (AJINOMOTO). Genome editing was conducted by using CRISPR/Cas9 technology (Institute of Immunology Co., Ltd.) (Table S1). Pluripotency markers, including OCT3/4, SSEA4, SOX2, and TRA‐1‐60 (antibodies were provided by BD Biosciences), were used to determine the stemness of iPSCs by flow cytometry. Chromosomal stability was evaluated through G‐band karyotyping (Tottori Bioscience Promotion Foundation). Sanger sequencing (CoMIT Omics Center, Osaka University) confirmed the presence of the missense mutation of c.508C>T in established iPSCs and correction in genome‐edited iPSCs. The primers are listed in Table S1.

### 2.3 Differentiation into CMs

iPSCs were seeded in iMatrix‐511 (Nippi)‐coated six‐well plates (Corning) at 2.5 × 10^4^ cells/well and cultured in AK02N medium (Ajinomoto) for 6 days. iPSCs were dissociated into single cells using 0.5× TrypLE select (Thermo Fisher Scientific) (1× TrypLE select diluted with 0.5 mM EDTA) and then seeded in a six‐well ultra‐low attachment plate (Corning) at 2 × 10^6^ cells/well in 1.5 mL/well StemPro‐34 medium (Thermo Fisher Scientific) containing 2 mM L‐glutamine (Thermo Fisher Scientific), 50 μL/mL ascorbic acid (AA; Sigma), 0.4 mM monothioglycerole (MTG; Sigma), 150 μg/mL transferrin (Wako), 10 μM ROCK inhibitor (Y‐27632; Fujifilm), 0.5% Matrigel (Corning), and 2 ng/mL BMP4 (R&D Systems) to form embryoid bodies (EBs). On day 1, 1.5 mL of StemPro‐34 medium containing 2 mM L‐glutamine, 50 μL/mL AA, 0.4 mM MTG, 150 μg/mL transferrin, 10 ng/mL bFGF (final 5 ng/mL), 24 or 12 ng/mL activin A (final 12 ng/mL for R170W iPSC line or 6 ng/mL for isogenic iPSC line), and 18 ng/mL BMP4 (final 10 ng/mL) was added into the well. On day 3, the EBs were rinsed once with Iscove's modified Dulbecco's medium (Thermo Fisher Scientific) and then cultured in 3 mL of StemPro‐34 medium containing 2 mM L‐glutamine, 50 μL/mL AA, 0.4 mM MTG, 150 μg/mL transferrin, 10 ng/mL vascular endothelial growth factor (VEGF; R&D Systems), 1 μM IWP‐3 (Stemgent), 0.6 μM dorsomorphin (Sigma), and 5.4 μM SB431542. On day 6, the medium was replaced with 3 mL of StemPro‐34 medium containing 2 mM L‐glutamine, 50 μL/mL AA, 0.4 mM MTG, 150 μg/mL transferrin, and 5 ng/mL VEGF. The EBs were then maintained in the same medium, with changes every 2–3 days. The plate was placed in a hypoxic environment (5% O_2_) for the first 8 days and then transferred to a normoxic environment.

### 2.4 Droplet digital PCR

Droplet digital PCR (ddPCR) (Bio‐Rad Laboratories) was performed according to the manufacturer's instructions. The primers and the probes used are listed in Table S1.

### 2.5 Western blot

Proteins were extracted using RIPA Buffer (Santa Cruz Biotechnology), and protein concentrations were measured using a NanoDrop 2000 spectrophotometer (Thermo Fisher Scientific). The extracted proteins were subsequently separated by SDS‐PAGE using 4%–20% Mini‐PROTEAN TGX Precast Gels (Bio‐Rad) and transferred to PVDF membranes (Bio‐Rad). Anti‐GAPDH (Santa Cruz Biotechnology, sc‐47724, 1:3000), anti‐TNNI1 (Abcam, ab203515, 1:2000), and anti‐TNNI3 (Proteintech, 21652‐1‐AP, 1:1000) were used as primary antibodies. Anti‐mouse antibody (CiteAB, NA9310V, 1:1000) and anti‐rabbit antibody (CiteAB, NA9340V, 1:10,000) were used as secondary antibodies.

### 2.6 Flow cytometric analysis

Differentiated EBs were dissociated into single cells using 0.5× TrypLE select for 15–20 min at 37°C and then suspended in PBS containing 10% FBS. The cells were analyzed and sorted as follows. For Troponin T staining, the cells were fixed and permeabilized using the BD Cytofix/Cytoperm™ Fixation/Permeabilization Kit (BD Bioscience) and then stained with purified mouse anti‐human cardiac Troponin T (Santa Cruz Biotechnology, sc‐20025, 1:200) and Goat anti‐Mouse Alexa Fluor 647 (Thermo Fisher Scientific, A21235, 1:300). The cells were detected using a FACSCanto II flow cytometer (BD Biosciences). For calcium transients, the cells were sorted using PE/Cy7‐conjugated anti‐SIRPa (BioLegend, 323808, 1:200) as a cardiomyocyte marker and APC‐labeled anti‐CD90 (BD Biosciences, 559869, 1:500), APC‐labeled anti‐human CD31 (BioLegend, 303116, 1:200), Alexa Fluor 647‐labeled anti‐CD49a (BioLegend, 328310, 1:200), and APC‐labeled anti‐CD140b (BioLegend, 323608, 1:200) as non‐cardiomyocyte lineage markers (Dubois et al., 2011; Miki et al., 2015). The cells were sorted using a FACSAria Fusion flow cytometer (BD Biosciences).

### 2.7 Immunocytochemistry

Dissociated cells were seeded onto a fibronectin‐coated chamber slide (Thermo Fisher Scientific) and cultured for 3–4 days. The cells were fixed with 4% paraformaldehyde (PFA) for 20 min, permeabilized PBS containing 0.1% Triton X‐100 for 15 min, and blocked with PBS containing 0.1% Triton X‐100 and 2% donkey serum at room temperature for 1 h. Then, the cells were stained with anti‐cardiac Troponin T (BD Pharmingen, 564766, 1:200) and anti‐ACTN2 (Abcam, ab68167, 1:200) overnight at 4°C. The following day, the cells were rinsed twice with PBS and stained with Alexa Fluor 488‐conjugated donkey anti‐mouse IgG (Thermo Fisher Scientific, 1:500) and Alexa Fluor 555‐conjugated donkey anti‐rabbit IgG (Thermo Fisher Scientific, 1:500) for 1 h at room temperature under dark conditions. The cells were rinsed twice with PBS and then stained with Hoechst (DOJINDO) for 5 min at room temperature under dark conditions. Fluorescence images were captured using a confocal laser scanning microscope (AX‐R, Nikon) and an inverted microscope (Ti2‐E, Nikon) equipped with an Apochromat Lambda S 60× oil objective lens (NA 1.40, Nikon) and a micro scanning stage to observe fluorescence images. Further analysis was conducted using NIS‐Elements software (Nikon). EHTs were fixed with 4% PFA for 2–3 h, rinsed with PBS, and immersed in 15% sucrose in PBS at 4°C overnight. The next day, the EHTs were placed in 30% sucrose in PBS and incubated at 4°C overnight. The EHTs were placed into cryomolds (Sakura Finetek Japan) and embedded using Tissue‐Tek Optimal Cutting Temperature compound (Sakura Finetek Japan). The EHTs were cryo‐sectioned into 5‐μm slices using a Leica microtome (Leica Biosystems).

### 2.8 Ca^2+^ oscillation

CMs, sorted by flow cytometry, were placed at 5 × 10^4^ cells/5 μL on the center of a fibronectin‐coated 35‐mm glass‐bottom dish (Iwaki), with the medium added 1 h later. The medium was changed every 2 days while the CMs were cultured. The CMs were treated with Calbryte™ 590 AM (AAT Bioquest) in accordance with the manufacturer's instructions. Fluorescence measurements were acquired with an AX‐R confocal microscope (Nikon) and an inverted microscope (Ti2‐E, Nikon) equipped with an Apochromat Lambda S 25× oil objective lens (Nikon) and a micro scanning stage to observe fluorescence images in living cells maintained at 37°C with a continuous supply of 95% air and 5% carbon dioxide by using a stage‐top incubator (STXG‐WSKMX‐SET, Tokai Hit) and analyzed using NIS‐Elements software (Nikon). The acquired data were further analyzed using MATLAB software (MathWorks).

### 2.9 Generation of EHT

The negative mold for the pillars was printed using an ABS filament (Zortrax) and a 3D printer (Zortrax). Then, PDMS was cast into the mold and incubated for 2 h at 60°C to form the pillar system. Ethylene oxide gas was used to sterilize the pillars, which were pretreated with a 5% solution of Pluronic F‐127 (Sigma). The hydrogel solution was prepared by mixing StemPro‐34 medium containing 2 mM L‐glutamine, 50 μg/mL AA, 0.4 mM MTG, 150 μg/mL transferrin, and 5 ng/mL VEGF with Fibrinogen (Sigma, final concentration 5 mg/mL), Matrigel (Corning, final concentration 5%), and aprotinin (Sigma, final concentration 5 μg/mL). To fabricate EHTs, differentiated EBs were dissociated into single cells using 0.5× TrypLE select for 15–20 min at 37°C, and the cells were then resuspended with the hydrogel solution at 20 × 10^6^ cells/mL. Thrombin (Sigma, final concentration 2.5 U/mL) was added to the cell suspension (200 μL), which was cast into the pillar system and incubated for 1 h at 37°C. The EHTs with pillars were subsequently transferred to a 24‐well plate and cultured in a medium containing 5 μg/mL aprotinin, with the medium being replaced every 2 days.

### 2.10 Force analysis of EHT

To analyze the force generation of EHTs, we initially recorded tissue contraction under 1 Hz stimulation using an HS All‐in‐One Fluorescence Microscope (BZ‐9000, KEYENCE). We then tabulated pillar deflection using custom‐made Python code and displacement measurements. We analyzed the acquired data using MATLAB software (MathWorks) to calculate contractile and relaxation forces.

By simulating a cantilever beam with a concentrated load at the free end, the force generated by EHTs was estimated. Tissue displacement corresponding to the amount of deflection served as the basis for the estimation. The amount of deflection, δ [mm], resulting from a concentrated load applied at the free end was calculated using the following equation:

$$\delta =\frac{{\mathrm{PL}}^{3}}{3\mathrm{EI}},$$

$$I=\frac{\pi }{64}d^{4},$$

where *P* is the concentrated force, *L* is the length of the pillar, *E* is the Young's modulus, and *d* is the diameter of the cantilever beam. In our experiments, *L* = 12 mm, *E* = 1.7 MPa, and *d* = 1.2 mm. EHTs were electrically stimulated at 1 Hz, and the behavior was recorded as a moving image using a digital video camera. The resolution of the captured images was 3.79 μm/pixel. We tracked the tissue across frames through the phase‐only correlation (POC) method (Takita et al., 2003) and measured its maximum deflection. POC is a template‐based image‐matching technique that detects subpixel accuracy in image registration between two images. This method demonstrates robustness in handling variations in image brightness owing to its reliance on phase information exclusively. As the deformation of the cell between adjacent frames is sufficiently small, POC proves to be effective in this task.

### 2.11 Transfection

To generate iPSC lines with stable TNNI3 expression, we constructed their vectors using the PiggyBack system (VectorBuilder). iPSCs were dissociated as described above and seeded on a laminin‐coated six‐well plate at 1 × 10^5^ cells/well in StemFit AK02N medium supplemented with 10 μM Y‐27632. The next day, the iPSCs were transfected with the target vector and the Super PiggyBac Transposase Expression Vector (SBI) using FuGENE® HD Transfection Reagent according to the manufacturer's protocol (Promega). After 2 days, the transfected iPSCs were sorted and seeded on laminin‐coated 6‐cm dishes at a low density of 300 or 600 cells/dish and cultured for 8–10 days to generate single clones. Then, we picked up some of them and established iPSC lines with the target gene integrated.

### 2.12 Statistical analysis

The data are presented as mean and SEM. Statistical analysis was performed using GraphPad Prism (GraphPad software). An unpaired two‐tailed Student's *t*‐test was used to analyze differences between the experimental groups.

## 3 RESULTS

### 3.1 Generation of disease‐specific iPSCs and isogenic iPSCs

**FIGURE 1:**
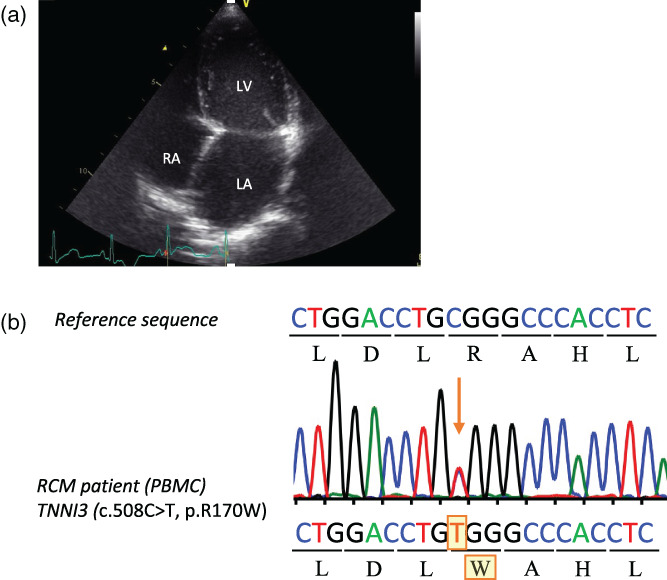
Patient with restrictive cardiomyopathy (RCM) harboring a heterozygous *TNNI3* mutation. (a) Echocardiogram of the proband with RCM. (b) Direct Sanger sequencing analysis of the *TNNI3* locus using genomic DNA obtained from the proband. PBMCs, peripheral blood mononuclear cells. Troponin I3 (TNNI3).

A 2‐year‐old boy was referred to our hospital due to congestive heart failure and diagnosed with severe idiopathic RCM. Echocardiography showed a preserved ejection fraction, normal left ventricular wall thickness, and a dilated left atrium (Figure 1a). At 3 years of age, the patient underwent left ventricular assist device implantation due to hemodynamic instability. Shortly after, a right ventricular assist device was added because of respiratory failure. At 4 years of age, heart transplantation was performed with no arrhythmic episodes from onset to transplantation. No relevant family history was reported. Whole exosome sequencing identified a heterozygous missense mutation in *TNNI3* (c.508C>T; p.Arg170Trp; R170W), which was further confirmed by Sanger sequencing (Figure 1b). This mutation was previously reported as a causative variant of pediatric RCM (Cimiotti et al., 2020). Patient‐specific iPSCs (R170W‐iPSCs) were generated from PBMCs isolated from the patient using the Cytotune‐iPS 2.0 Sendai reprogramming Kit. Evaluation of the generated iPSCs confirmed they were positive for OCT3/4, SSEA4, SOX2, and TRA‐1‐60, and they had a normal karyotype (Figure S1a,b). Subsequent Sanger sequencing of genomic DNA from R170W‐iPSCs was conducted to assess genetic mutations in the *TNNI3* locus (Figure S1c). We also generated an isogenic cell line with a corrected mutation to the pseudo‐wild type (Isogenic‐iPSCs) using the CRISPR/Cas9 system to evaluate the pathological phenotype of the R170W mutation in the same genetic background. The generated Isogenic‐iPSCs were positive for pluripotency markers, possessed a normal karyotype, and had corrected sequences (Figure S1d–f).

### 3.2 Differentiation of iPSCs into CMs

**FIGURE 2:**
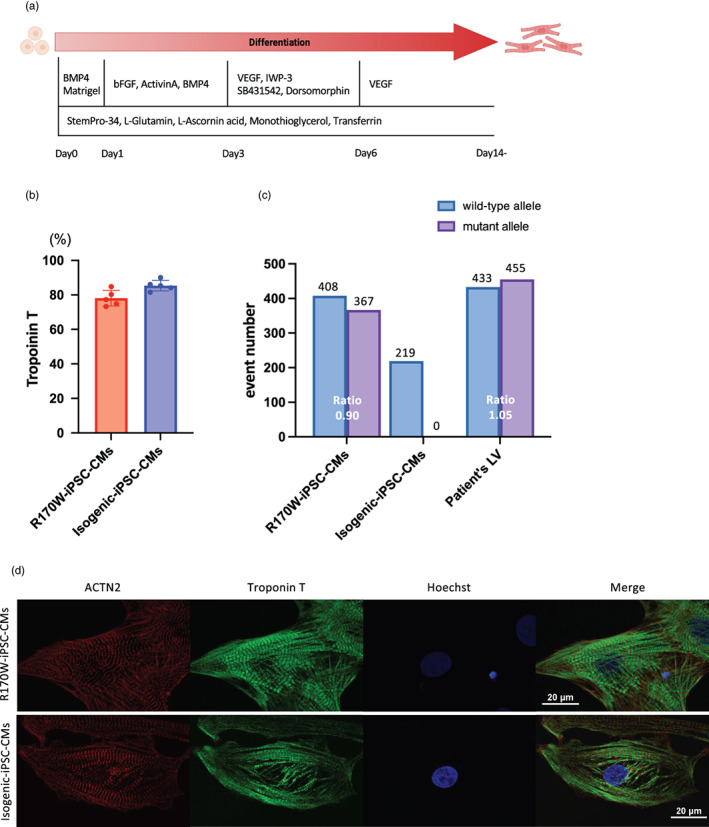
Characterization of R170W induced pluripotent stem cells (iPSCs) and Isogenic‐iPSCs. (a) Time course of monolayered differentiation into cardiomyocytes. (b) Troponin T‐positive ratio in flow cytometric (FACS) analysis of R170W‐iPSC‐CMs (*n* = 5) and Isogenic‐iPSC‐CMs (*n* = 5). Data are presented as the mean ± SEM. (c) Results of droplet digital PCR analysis using cDNA samples obtained from R170W‐iPSC‐CMs, Isogenic‐iPSC‐CMs, and a fresh frozen left ventricle (LV) sample from the proband. (d) Representative immunofluorescence images of R170W‐ and Isogenic‐iPSC‐CMs stained with anti‐ACTN2 (red), anti‐Troponin T (green), and Hoechst (blue). Scale bar: 20 μm. VEGF, vascular endothelial growth factor. actinin alpha 2 (ACTN2).

R170W‐iPSCs and Isogenic‐iPSCs were differentiated into CMs (Figure 2a), and spontaneous beating was observed in these iPSC‐CMs around day 8 after differentiation induction. To analyze differentiation efficiency, we used flow cytometry using an anti‐Troponin T antibody on day 14 (Figure 2b). To examine the number of transcripts from the wild‐type and mutant (R170W) alleles, we performed ddPCR analysis using cDNA synthesized from RNA samples of R170W‐iPSC‐CMs, Isogenic‐iPSC‐CMs, and a cardiac sample obtained from the patient's left ventricle. The ddPCR results revealed that R170W‐iPSC‐CMs and the cardiac sample contained comparable amounts of transcripts from the wild‐type and mutant alleles (Figure 2c). In contrast, Isogenic‐iPSC‐CMs featured no transcripts of the mutant allele (Figure 2c). Next, we examined R170W‐iPSC‐CMs and Isogenic‐iPSC‐CMs using immunofluorescence analysis, which showed no observable variations in the sarcomere structure (Figure 2d).

### 3.3 R170W‐iPSC‐CMs exhibited prolonged diastolic time

**FIGURE 3:**
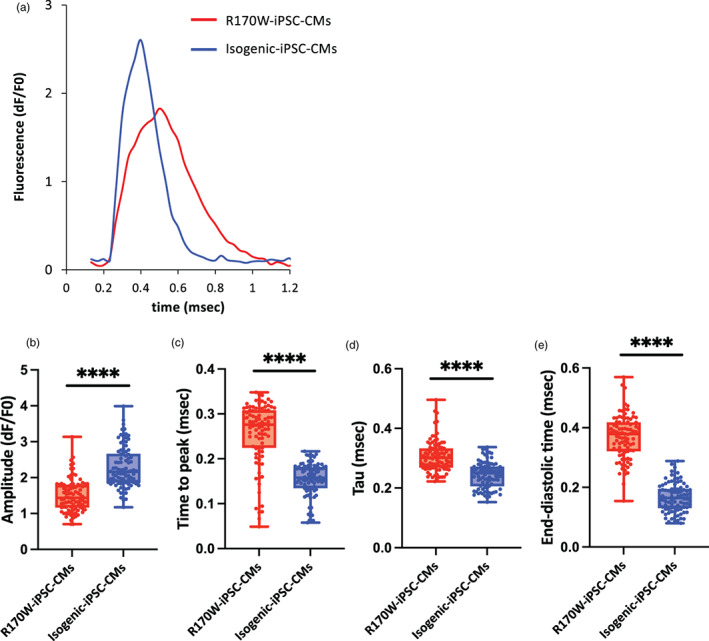
Ca^2+^ transients of R170W induced pluripotent stem cells (iPSCs) and Isogenic‐iPSCs. (a) Representative recordings of the fluorescence signal at a pacing rate of 1 Hz. (b–e) Amplitude (b), time to peak (c), tau (d), and end‐diastolic time (e) measured using high‐throughput fluorescence imaging at 1.0 Hz pacing (10 waves on average per region of interest (ROI)). *n* = 90 (R170W‐iPSC‐CMs) and *n* = 90 (Isogenic‐iPSC‐CMs) over three independent experiments. Boxes represent the 25th and 75th percentiles; whiskers represent the minimum and maximum ranges; horizontal lines indicate the median values. Statistical analyses were performed using unpaired two‐tailed Student's *t*‐tests. ****p < .0001. iPSC‐CMs, iPSC‐derived cardiomyocytes.

To investigate the previously reported phenotypic alterations in RCM associated with *TNNI3* mutations (Davis et al., 2007; Yumoto et al., 2005), we evaluated the intracellular Ca^2+^ kinetics in iPSC‐CMs using Calbryte™ 590 AM as a calcium indicator (Videos S1 and S2). iPSC‐CMs were electrically stimulated at 1 Hz during the video recording. Figure 3a shows representative recordings of the fluorescence signal waveforms in R170W‐ and Isogenic‐iPSC‐CMs. R170W‐iPSC‐CMs exhibited significantly decreased amplitudes, along with a prolonged time to peak (Figure 3b,c). The Ca^2+^ tau, the time for the signal to decrease from 100% to 20%, which represents Ca^2+^ reuptake efficacy during relaxation, was significantly prolonged in R170W‐iPSC‐CMs (Figure 3d). Furthermore, the end‐diastolic time, the time for the signal to decrease from 20% to 0%, was also significantly prolonged in R170W‐iPSC‐CMs (Figure 3e). These results indicate that the *TNNI3* R170W mutation impaired relaxation function and caused abnormal cytosolic Ca^2+^ flux in iPSC‐CMs.

### 3.4 Contraction and relaxation kinetics of R170W‐ and Isogenic‐EHTs

**FIGURE 4:**
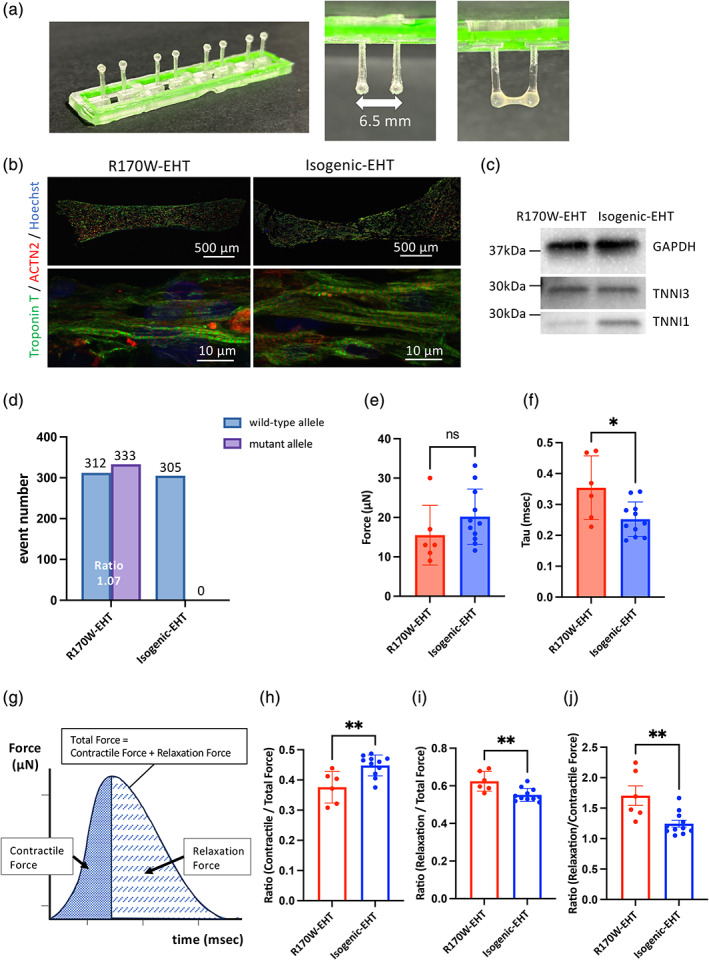
Generation of engineered heart tissues (EHTs) using R170W‐iPSCs and isogenic induced pluripotent stem cells (iPSCs). (a) Tissue pillars. The EHT pillar spacing was 6.5 mm. Side view of tissue attaching to the pillar after 2 weeks. (b) Representative immunofluorescence images of R170W‐EHT and Isogenic‐EHT stained with anti‐Troponin T (green), anti‐ACTN2 (red), and Hoechst (blue). (c) Representative western blot images of R170W‐EHT and Isogenic‐EHT lysates 14 days after EHT generation using the indicated antibodies. The molecular weight ladder is displayed on the left. (d) Results of droplet digital PCR analysis using cDNA samples obtained from R170W‐EHTs and Isogenic‐EHTs. (e) Measurement of the force at maximum pillar deflection of the EHT 2 weeks after tissue fabrication (*n* = 6–11 tissues per group). Data are presented as the mean ± SEM. Statistical analysis was performed using unpaired two‐tailed Student's *t*‐tests. (f) Decay tau of relaxation force. Data are presented as the mean ± SEM. Statistical analysis was performed using unpaired two‐tailed Student's *t*‐tests. *p < .05. (g) Schematic diagram of the force waveform obtained using video microscopy. (h–j) Calculated ratios of contractile force/total force (h), relaxation force/total force (i), relaxation force/contractile force (j) of EHT 2 weeks after tissue fabrication (*n* = 6–11 tissues per group). Data are presented as the mean ± SEM. Statistical analyses were performed using unpaired two‐tailed Student's *t*‐tests. **p < .01. actinin alpha 2 (ACTN2).

Three‐dimensional engineered tissues have significantly contributed to disease modeling and drug discovery by overcoming the immaturity of iPSC‐CMs. Studies using these tissues have reported impaired myocardial relaxation (Wang et al., 2023a) as discussed in previous works (Campostrini et al., 2021; Ronaldson‐Bouchard et al., 2018). To investigate the contractile dynamics in the *TNNI3* mutant RCM model, we fabricated EHTs using R170W‐iPSC‐CMs (R170W‐EHT) and Isogenic‐iPSC‐CMs (Isogenic‐EHT) (Figure 4a). The EHTs initiated contractions after approximately 8 days of cultivation (Videos S3 and S4). Subsequently, on day 14, we recorded pillar deflection data and conducted immunocytochemistry and RNA and protein extraction. Immunofluorescence imaging of the entire EHT revealed no noteworthy difference in sarcomere structures between R170W‐EHT and Isogenic‐EHT (Figure 4b). As the TNNI isoform switch from TNNI1 to TNNI3 in CMs is one of the significant indicators of cardiac maturation (Bedada et al., 2014), we performed western blot analysis to determine the TNNI3 protein expression levels in both EHTs (Figure 4c). This indicated that EHT fabrication promotes cardiac maturation. In addition, the amounts of transcripts from the wild‐type and R170W alleles of *TNNI3* in EHTs were evaluated by ddPCR. It was found that the ratio of transcript levels in R170W‐EHT was almost equal to 1:1, consistent with the transcript levels found in differentiated CMs (Figures 2c and 4d). These findings suggest that the relative transcript levels between alleles remain constant during maturation, suggesting that the wild‐type and mutant proteins could be present at the same levels.

To assess the kinetics of EHT contraction, we used video microscopy to track one‐dimensional pillar deflection under 1 Hz stimulation (Videos S5 and S6). The distance of the pillar head movement resulting from EHT beating was gauged, and the force (N) was derived by calculating the distance of movement along with the material data of the pillar. The force formula relies solely on the change in the pillar's amount as the only parameter. The force at the maximum pillar deflection is shown in Figure 4e. No difference in force was observed between R170W‐EHT and Isogenic‐EHT. On the other hand, the force decay tau, the time for the pillar deflection to decrease from 100% to 20% during relaxation, was significantly longer in R170W‐EHT than in Isogenic‐EHT (Figure 4f). Using the force waveform calculated from the pillar displacement, we defined the force during systole as the contractile force and the force during diastole as the relaxation force (Figure 4g). As the total force is expressed as the area of the waveform (Figure 4g), the relative proportions of contractile and relaxation forces within the total force applied during a cycle can be calculated. The contractile force is the force that displaces the pillars during tissue contraction, the relaxation force is the residual force that causes pillar deflection during maximum relaxation, and the total force is the sum of the contractile and relaxation forces. The ratio of contractile force to total force was lower and the ratio of relaxation force to total force was higher in R170W‐EHT than in Isogenic‐ETH (Figure 4h,i). Additionally, the ratio of relaxation force to contractile force was significantly higher in R170W‐EHT than in Isogenic‐EHT (Figure 4j). Collectively, by comparing the contractile and relaxation forces and their ratio in vitro, these data suggest that our EHT model reflects the clinical manifestation of RCM with diastolic dysfunction.

### 3.5 Improvement of relaxation impairment of R170W‐EHTs by overexpression of TNNI3

**FIGURE 5:**
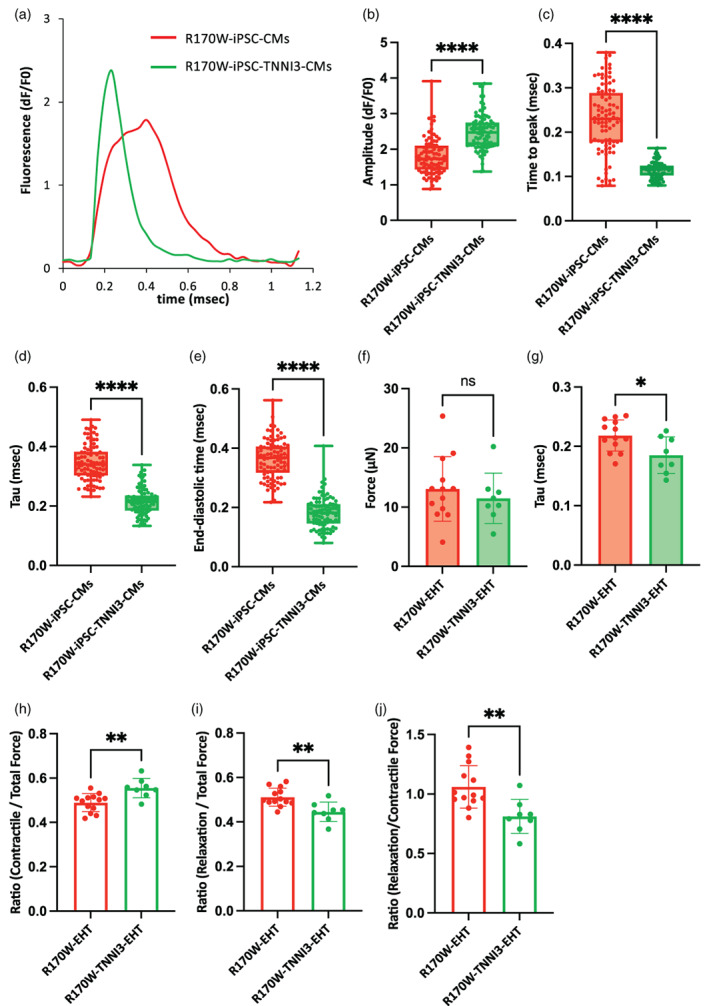
Overexpression of TNNI3 restored impaired relaxation in cardiomyocytes (CMs) and engineered heart tissues (EHTs). (a) Representative recordings of the fluorescence signal at a pacing rate of 1 Hz. (b–e) Amplitude (b), time to peak (c), tau (d), and end‐diastolic time (e) measured using high‐throughput fluorescence imaging at 1.0 Hz pacing (10 waves on average per region of interest). *n* = 90 (R170W‐iPSC‐CMs) and *n* = 90 (R170W‐iPSC‐TNNI3‐CMs) over three independent experiments. Boxes represent the 25th and 75th percentiles; whiskers represent the minimum and maximum ranges; horizontal lines indicate the median values. Statistical analyses were performed using unpaired two‐tailed Student's *t*‐tests. ****p < .0001. (f) Measurement of the force at maximum pillar deflection of the EHT 2 weeks after tissue fabrication (*n* = 8–13 tissues per group). Data are presented as the mean ± SEM. Statistical analysis was performed using unpaired two‐tailed Student's *t*‐tests. (g) Decay tau of relaxation force. Data are presented as the mean ± SEM. Statistical analysis was performed using unpaired two‐tailed Student's *t*‐tests. *p < .05. (h–j) Calculated ratios of contractile force/total force (h), relaxation force/total force (i), and relaxation force/contractile force (j) of the EHT 2 weeks after tissue fabrication (*n* = 8–13 tissues per group). Data are presented as the mean ± SEM. Statistical analyses were performed using unpaired two‐tailed Student's *t*‐tests. **p < .01. Troponin I3 (TNNI3).

Based on our findings that R170W‐EHTs expressing 50% of the R170W mutant protein of TNNI3 displayed a relaxation impairment phenotype when compared to Isogenic‐EHTs lacking expression of the mutant protein, we hypothesized that overexpression of the normal TNNI3 proteins in R170W‐EHTs would restore the phenotype. To evaluate this hypothesis, we generated an iPSC line that consistently expressed normal TNNI3 proteins (Figure S2a) and performed cardiac differentiation similarly. We observed beating CMs (R170W‐iPSC‐TNNI3‐CMs) that consistently expressed TNNI3‐EGFP (Video S7). We confirmed the differentiation efficiency by Troponin T flow cytometry (Figure S2b) and demonstrated the forced expression of normal *TNNI3* mRNA by ddPCR (Figure S2c). Western blot analysis showed the expression of TNNI3 in both EHTs (Figure S2d). To assess the intracellular Ca^2+^ kinetics in both R170W‐iPSC‐CMs and R170W‐TNNI3‐iPSC‐CMs, we used Calbryte™ 590 AM as the calcium indicator (Video S8) and recorded the calcium fluorescence under 1 Hz stimulation (Figure 5a). The amplitude and time to peak were significantly improved in R170W‐iPCS‐TNNI3‐CMs (Figure 5b,c). Furthermore, compared with R170W‐iPSC‐CMs, Ca^2+^ tau and end‐diastolic time were improved (Figure 5d,e).

The contraction kinetics of EHT were evaluated as previously described. The force at the maximum pillar deflection showed no difference between R170W‐EHT and R170W‐TNNI3‐EHT (Figure 5f). Significant differences were found in force tau, the time for EHT to relax from 100% to 20% during relaxation (Figure 5g). In R170W‐TNNI3‐EHT, the ratio of contractile force to total force was significantly higher and the ratio of relaxation force to total force was significantly lower than in R170W‐EHT. The ratio of relaxation force to contractile force was also significantly lower in R170W‐TNNI3‐EHT. This was similar to the results of the comparison with Isogenic‐EHT. These data demonstrate that overexpression of normal TNNI3 ameliorates the relaxation impairment phenotype exhibited by R170W‐iPSC‐CMs and ‐EHTs. Taken together, these findings suggest the potential therapeutic effectiveness of overexpression of normal TNNI3 to improve relaxation impairment in RCM caused by the R170W mutation.

## 4 DISCUSSION

In this study, we differentiated iPSCs derived from a patient with RCM harboring the R170W mutation into CMs. We then generated the EHT, a construct analogous to mature cardiac tissue characterized by elevated TNNI3 expression. Our evaluation system, which measured contractile dynamics, was designed in‐house. The deflection of the pillar was the sole variable in this system, and it was proportional to the force produced by the EHTs. Thus, the force waveform could be extracted from the video of the pillar movement. Using this waveform, the force up to the pillar's maximum contraction was identified as the contractile force, and the force up to its return to the original position was identified as the relaxation force. Compared to the Isogenic‐EHT, the R170W‐EHT exhibited a significantly greater relaxation force relative to the contractile force (Figure 4j). The results show that the relaxation force was higher in R170W‐EHT than in Isogenic‐EHT. This may indicate diastolic dysfunction of RCM in clinical practice. Additionally, considering that this rate was improved in R170W‐TNNI3‐EHT (Figure 5j), this evaluation system may be a useful model for evaluating relaxation impairment in vitro. A limitation of this evaluation system is that the forces are only measured in a single longitudinal direction of the pillar, which may affect the accuracy of our model in representing diastolic dysfunction in the patient's heart. Furthermore, the maturation level of EHTs cannot be quantitatively assessed. Western blot analysis showed that the ratio of TNNI3 to TNNI1 is higher in R170W‐EHT than in Isogenic‐EHT (Figure 4c). Although it is generally accepted that cardiac maturation in iPSC‐CMs contributes to increased contractility and shortening of Ca^2+^ tau (Pioner et al., 2022), we observed no difference in the force at the maximum pillar deflection (Figure 4e) and shorter tau in Isogenic‐EHT (Figure 4f). We also observed significantly prolonged Ca^2+^ tau in R170W‐iPSC‐CMs (Figure 3d). These results suggest that the prolonged force tau in R170W‐EHT is due to maturation. The patient with the R170W mutation was a severe case with early onset of symptoms, such as heart failure at less than 1 year of age. The same mutation has been reported to cause severe RCM (Cimiotti et al., 2020). These findings suggest that this mutation causes an early‐onset phenotype even in iPSC‐CMs, which are relatively immature.

Nonetheless, we found significant differences between the disease and gene correction models, which highlight the importance of our findings. In clinical practice, evaluation of diastolic dysfunction in RCM involves determining ventricular pressure through cardiac catheterization (Rapezzi et al., 2022). However, there is currently no methodology available for direct quantification of diastolic dysfunction in vitro. There have been multiple studies on systems that measure force directly using three‐dimensional cardiac tissues in an in vitro model without partitioning the force into systolic and diastolic phases (Wang, Nash, et al., 2023; Zhao et al., 2019). In their RCM model, Wang et al. used ECT from a patient with RCM harboring an *FLNC* mutation to measure both direct force and calcium kinetics. Their results indicated that the tissue exhibited relaxation impairment (Wang et al., 2023a). Our study lends support to this paradigm by confirming a comparable phenotype in an RCM case with a *TNNI3* mutation. Consequently, our findings suggest that the clinical phenotype of diastolic dysfunction in RCM may manifest as an augmented diastolic force of EHT in vitro, regardless of the location of the mutation, and that our EHT system may serve as a model to study diastolic dysfunction.

Studies using skinned fibers with the *TNNI3* R170W mutation have demonstrated an increase in calcium sensitivity resulting from the R170W mutation (Cimiotti et al., 2020). Furthermore, at the in vivo level, it has also been reported that prolonged Ca^2+^ tau occurs in *TNNI3* mutant RCM mice (Davis et al., 2007; Du et al., 2008; Wen et al., 2009). Given that the rate of calcium fluorescence can be used as a surrogate index of myocyte contraction–relaxation kinetics (Wang et al., 2023a), prolonged Ca^2+^ tau may represent prolonged myocardial relaxation time, which may lead to impaired relaxation. In fact, prolonged Ca^2+^ tau is believed to play an important role in the development of RCM in *TNNI3* mutant myocardium (Li et al., 2010). A previous study reported a correlation between the degree of calcium sensitivity and the degree of relaxation impairment (Yumoto et al., 2005). In vitro calcium imaging is a crucial experimental system for assessing the clinical phenotype of RCM. Similar to these previous studies, our R170W‐iPSC‐CMs demonstrated prolonged Ca^2+^ tau in the Ca^2+^ oscillation experimental system, along with a decreased amplitude and prolonged time to peak (Figure 3b,c). Both decreased amplitude and prolonged time to peak have been reported in heart failure with impaired contractility (Gómez et al., 1997). Although the diagnostic criteria for RCM include diastolic dysfunction with preserved contractility, the progression of heart failure and severe RCM leads to gradual contractile dysfunction development. In the present study, the ratio of contractile force to total force was also decreased in R170W‐EHT (Figures 4h and 5h). This result may indicate subclinical systolic dysfunction in RCM. The exact molecular mechanism by which *TNNI3* mutations increase Ca^2+^ sensitivity is currently unknown. However, the prolonged Ca^2+^ transient decay time observed in skinned myocardium after replacement of C‐terminally truncated Troponin I (Tachampa et al., 2008) suggests that C‐terminal mutations in TNNI3 might play a role in the elevated frequency of severe RCM by affecting Troponin C or modifying subsequent EC coupling. Interestingly, R170W‐iPSC‐CMs exhibited a significant prolongation of the calculated end‐diastolic time (a reduction in dF/F0 from 20% to 0%) (Figure 3e). This report presents the first examination of end‐diastolic time during the cardiac cycle at a dF/F0 level of 20%–0%. Although tau has been used for evaluating diastolic time in vitro, it has not been focused on end‐diastole. Clinically, end‐diastolic pressure is measured directly during cardiac catheterization to evaluate diastolic dysfunction. However, there is currently no in vitro evaluation system for pressure measurement. Using the in vitro system, we evaluated the time of end‐diastole and found a significant difference between R170W‐iPSC‐CMs and Isogenic‐iPSC‐CMs (Figure 3e). It is also interesting to note that the decay time from 20% to 0% was longer for R170W‐iPSC‐CMs and shorter for Isogenic‐iPSC‐CMs compared to tau (from 100% to 20%) (Figure 3d,e). This suggests that the end‐diastolic state may be a more sensitive reflection of pathology. As end‐diastole is the moment of the highest pressure load on the left ventricle, evaluation of end‐diastole may be important for future models to evaluate diastolic dysfunction. In the future, more detailed duration settings and interoperability with in vivo models are required.

A correlation between the phenotype and the percentage of mutant mRNAs and proteins has been previously reported in cardiomyopathy. In familial hypertrophic cardiomyopathy, the ratio of mutated *MYH7* mRNA and protein correlates with the reported severity of the clinical outcome (Tripathi et al., 2011). In RCM, it has been reported that TNNI3 proteins harboring the R193H mutation are more effectively incorporated into sarcomeres (Davis et al., 2007). This leads to dose‐dependent effects on both basal and dynamic contractile function. Our findings using Isogenic‐iPSC‐CMs and ‐EHT also showed tau shortening and a reduced ratio of relaxation force to contractile force (Figure 4f,i,j), highlighting the potential of CRISPR system‐based gene therapy for repair of mutated sites through the use of base and prime editors (Anzalone et al., 2020; Chai et al., 2023). Another promising approach to ameliorate cardiomyopathy is the delivery of genes with beneficial functions. For example, a single administration of an AAV9 vector harboring the *MYBPC3* gene (AAV9‐MYBPC3) in a homozygous *MYBPC3* mutant mouse model of neonatal hypertrophic cardiomyopathy prevented the development of cardiac hypertrophy during 34 weeks of observation and resulted in elevated MYBPC3 mRNA and protein levels in a dose‐dependent manner (Mearini et al., 2014). Furthermore, administration of an AAV9 vector harboring the *BAG3* gene (AAV9‐BAG3) in a mouse model of myocardial infarction resulted in the restoration of cardiac function and myofilament turnover (Martin et al., 2021). Moreover, intravenous administration of AAV9‐BAG3 prevented the development of a reduced ejection fraction in *Bag3* haplo‐deficient mice (Myers et al., 2018). These advancements have paved the way for gene therapy of the genes encoding MYBPC3 and BAG3, for which phase I clinical trials are planned. Therapeutic approaches that induce overexpression of normal proteins are promising for clinical applications due to their feasibility. We speculated that overexpression of normal TNNI3 in our R170W‐iPSCs would ameliorate relaxation impairment as well. The results indicated that normal *TNNI3* mRNAs were highly expressed in R170W‐iPSC‐TNNI3‐CMs while mutant *TNNI3* mRNAs were undetectable (Figure S2c), resulting in improved relaxation impairment in both calcium kinetics and contraction kinetics of EHT (Figure 5). This shows the possibility of gene therapy using a single modality for RCM patients with *TNNI3* mutations, regardless of the mutation site.

Notable results of our study demonstrated that R170W‐iPSC‐CMs and ‐EHT exhibited prolonged relaxation time and relaxation impairment, and an evaluation model was established in vitro. Furthermore, gene correction and overexpression of *TNNI3* improved relaxation impairment in iPSC‐CMs and ‐EHT. Thus, our model could provide insight into the pathogenesis of RCM and support the development of therapeutic strategies.

## AUTHOR CONTRIBUTIONS

M.H., K.M., T.K., M.T., H.I., S.H., Y.S., and S.M. conceived and designed the project. M.H., K.M., I.T.S., Y.H., M.T., K.K., E.I., and M.T. performed the experimental work. M.H., K.M., and S.M. interpreted the data and wrote the manuscript. All the authors discussed the results.

## Supporting information


**Figure S1.** Generation of R170W‐iPSCs and Isogenic‐iPSCs. (a) R170W‐iPSC flow cytometry analysis of the pluripotency markers OCT3/4, SOX2, SSEA4, and TRA‐1‐60. Almost all cells were positive for these markers. (b) The karyotype of R170W‐iPSCs showed a normal male pattern. (c) Direct Sanger sequencing analysis of the R170W‐iPSCs using genomic DNA obtained from the cells.
(d) Isogenic‐iPSC flow cytometry analysis of the pluripotency markers OCT3/4, SOX2, SSEA4, and TRA‐1‐60. Almost all cells were positive for these markers.
(e) The karyotype of Isogenic‐iPSCs showed a normal male pattern.
(f) Direct Sanger sequencing analysis of the Isogenic‐iPSCs using genomic DNA obtained from the cells.
**Figure S2.** Generation of R170W‐TNNI3‐iPSCs.
(a) Schematic diagram of transfection of R170W‐iPSCs using the *TNNI3* gene vector.
(b) Troponin T‐positive ratio in flow cytometric analysis of R170W‐iPSC‐TNNI3‐CMs (*n* = 5). Data are presented as the mean ± SEM.
(c) Results of droplet digital PCR analysis using cDNA samples obtained from R170W‐iPSC‐CMs and R170W‐iPSC‐TNNI3‐CMs.
(d) Representative western blot images of R170W‐EHT and R170W‐TNNI3‐EHT lysates 14 days after EHT generation using the indicated antibodies. The molecular weight ladder is shown on the left.
**Table S1.** Sequence information.

**Video S1.** Representative video of R170W‐iPSC‐CMs beating using Calbryte™ 590 AM as the calcium indicator under 1 Hz stimulation.

**Video S2.** Representative video of Isogenic‐iPSC‐CMs beating using Calbryte™ 590 AM as the calcium indicator under 1 Hz stimulation.

**Video S3.** Representative video of R170W‐EHT and Isogenic‐EHT paced at 1 Hz after 2 weeks of culture.

**Video S4.** Representative video of Isogenic‐EHT paced at 1 Hz after 2 weeks of culture.

**Video S5.** Representative video of R170W‐EHT using video microscopy to track pillar deflection under 1 Hz stimulation.

**Video S6.** Representative video of Isogenic‐EHT using video microscopy to track pillar deflection under 1 Hz stimulation.

**Video S7.** Beating of R170W‐iPSC‐TNNI3‐CMs with EGFP expression.

**Video S8.** Representative video of R170W‐iPSC‐TNNI3‐CMs beating using Calbryte™ 590 AM as the calcium indicator under 1 Hz stimulation.

## References

[dgd12909-bib-0001] Anderson, H. N. , Cetta, F. , Driscoll, D. J. , Olson, T. M. , Ackerman, M. J. , & Johnson, J. N. (2018). Idiopathic restrictive cardiomyopathy in children and Young adults. The American Journal of Cardiology, 121, 1266–1270. 29526277 10.1016/j.amjcard.2018.01.045

[dgd12909-bib-0002] Anzalone, A. V. , Koblan, L. W. , & Liu, D. R. (2020). Genome editing with CRISPR‐Cas nucleases, base editors, transposases and prime editors. Nature Biotechnology, 38, 824–844. 10.1038/s41587-020-0561-9 32572269

[dgd12909-bib-0003] Bedada, F. B. , Chan, S. S. , Metzger, S. K. , Zhang, L. , Zhang, J. , Garry, D. J. , Kamp, T. J. , Kyba, M. , & Metzger, J. M. (2014). Acquisition of a quantitative, stoichiometrically conserved ratiometric marker of maturation status in stem cell‐derived cardiac myocytes. Stem Cell Reports, 3, 594–605. 25358788 10.1016/j.stemcr.2014.07.012 PMC4223713

[dgd12909-bib-0004] Bellin, M. , Marchetto, M. C. , Gage, F. H. , & Mummery, C. L. (2012). Induced pluripotent stem cells: The new patient? Nature Reviews. Molecular Cell Biology, 13, 713–726. 23034453 10.1038/nrm3448

[dgd12909-bib-0005] Campostrini, G. , Windt, L. M. , Van Meer, B. J. , Bellin, M. , & Mummery, C. L. (2021). Cardiac tissues from stem cells: New routes to maturation and cardiac regeneration. Circulation Research, 128, 775–801. 33734815 10.1161/CIRCRESAHA.121.318183 PMC8410091

[dgd12909-bib-0006] Chai, A. C. , Cui, M. , Chemello, F. , Li, H. , Chen, K. , Tan, W. , Atmanli, A. , McAnally, J. R. , Zhang, Y. , Xu, L. , Liu, N. , Bassel‐Duby, R. , & Olson, E. N. (2023). Base editing correction of hypertrophic cardiomyopathy in human cardiomyocytes and humanized mice. Nature Medicine, 29, 401–411. 10.1038/s41591-022-02176-5 PMC10053064 36797478

[dgd12909-bib-0007] Cimiotti, D. , Fujita‐Becker, S. , Möhner, D. , Smolina, N. , Budde, H. , Wies, A. , Morgenstern, L. , Gudkova, A. , Sejersen, T. , Sjöberg, G. , Mügge, A. , Nowaczyk, M. M. , Reusch, P. , Pfitzer, G. , Stehle, R. , Schröder, R. R. , Mannherz, H. G. , Kostareva, A. , & Jaquet, K. (2020). Infantile restrictive cardiomyopathy: cTnI‐R170G/W impair the interplay of sarcomeric proteins and the integrity of thin filaments. PLoS ONE, 15, e0229227. 32182250 10.1371/journal.pone.0229227 PMC7077804

[dgd12909-bib-0008] Conway, J. , Min, S. , Villa, C. , Weintraub, R. G. , Nakano, S. , Godown, J. , Tatangelo, M. , Armstrong, K. , Richmond, M. , Kaufman, B. , Lal, A. K. , Balaji, S. , Power, A. , Baez Hernandez, N. , Gardin, L. , Kantor, P. F. , Parent, J. J. , Aziz, P. F. , Jefferies, J. L. , … Mital, S. (2023). The prevalence and Association of Exercise Test Abnormalities with Sudden Cardiac Death and Transplant‐Free Survival in childhood hypertrophic cardiomyopathy. Circulation, 147, 718–727. 36335467 10.1161/CIRCULATIONAHA.122.062699 PMC9977414

[dgd12909-bib-0009] Davis, J. , Wen, H. , Edwards, T. , & Metzger, J. M. (2007). Thin filament disinhibition by restrictive cardiomyopathy mutant R193H troponin I induces Ca2+−independent mechanical tone and acute myocyte remodeling. Circulation Research, 100, 1494–1502. 17463320 10.1161/01.RES.0000268412.34364.50

[dgd12909-bib-0010] den Boer, S. L. , van Osch‐Gevers, M. , van Ingen, G. , du Marchie Sarvaas, G. J. , van Iperen, G. G. , Tanke, R. B. , Backx, A. P. C. M. , ten Harkel, A. D. J. , Helbing, W. A. , Delhaas, T. , Bogers, A. J. J. C. , Rammeloo, L. A. J. , & Dalinghaus, M. (2015). Management of children with dilated cardiomyopathy in The Netherlands: Implications of a low early transplantation rate. The Journal of Heart and Lung Transplantation, 34, 963–969. 25840505 10.1016/j.healun.2015.01.980

[dgd12909-bib-0011] Du, J. , Liu, J. , Feng, H. Z. , Hossain, M. M. , Gobara, N. , Zhang, C. , Li, Y. , Jean‐Charles, P. Y. , Jin, J. P. , & Huang, X. P. (2008). Impaired relaxation is the main manifestation in transgenic mice expressing a restrictive cardiomyopathy mutation, R193H, in cardiac TnI. American Journal of Physiology Heart and Circulatory Physiology, 294, H2604–H2613. 18408133 10.1152/ajpheart.91506.2007 PMC2769498

[dgd12909-bib-0012] Dubois, N. C. , Craft, A. M. , Sharma, P. , Elliott, D. A. , Stanley, E. G. , Elefanty, A. G. , Gramolini, A. , & Keller, G. (2011). SIRPA is a specific cell‐surface marker for isolating cardiomyocytes derived from human pluripotent stem cells. Nature Biotechnology, 29, 1011–1018. 10.1038/nbt.2005 PMC4949030 22020386

[dgd12909-bib-0013] Feric, N. T. , & Radisic, M. (2016). Maturing human pluripotent stem cell‐derived cardiomyocytes in human engineered cardiac tissues. Advanced Drug Delivery Reviews, 96, 110–134. 25956564 10.1016/j.addr.2015.04.019 PMC4635107

[dgd12909-bib-0014] Fujiwara, Y. , Miki, K. , Deguchi, K. , Naka, Y. , Sasaki, M. , Sakoda, A. , Narita, M. , Imaichi, S. , Sugo, T. , Funakoshi, S. , Nishimoto, T. , Imahashi, K. , & Yoshida, Y. (2023). ERRgamma agonist under mechanical stretching manifests hypertrophic cardiomyopathy phenotypes of engineered cardiac tissue through maturation. Stem Cell Reports, 18, 2108–2122. 37802074 10.1016/j.stemcr.2023.09.003 PMC10679535

[dgd12909-bib-0015] Gómez, A. M. , Valdivia, H. H. , Cheng, H. , Lederer, M. R. , Santana, L. F. , Cannell, M. B. , McCune, S. A. , Altschuld, R. A. , & Lederer, W. J. (1997). Defective excitation‐contraction coupling in experimental cardiac hypertrophy and heart failure. Science, 276, 800–806. 9115206 10.1126/science.276.5313.800

[dgd12909-bib-0016] Ishida, H. , Narita, J. , Ishii, R. , Suginobe, H. , Tsuru, H. , Wang, R. , Yoshihara, C. , Ueyama, A. , Ueda, K. , Hirose, M. , Hashimoto, K. , Nagano, H. , Kogaki, S. , Kuramoto, Y. , Miyashita, Y. , Asano, Y. , & Ozono, K. (2023). Clinical outcomes and genetic analyses of restrictive cardiomyopathy in children. Circulation: Genomic and Precision Medicine, 16, 382–389. 37377035 10.1161/CIRCGEN.122.004054

[dgd12909-bib-0017] Jaferzadeh, K. , Rappaz, B. , Kim, Y. , Kim, B. K. , Moon, I. , Marquet, P. , & Turcatti, G. (2023). Automated dual‐mode cell monitoring to simultaneously explore calcium dynamics and contraction‐relaxation kinetics within drug‐treated stem cell‐derived Cardiomyocytes. ACS Sensors, 8, 2533–2542. 37335579 10.1021/acssensors.3c00073

[dgd12909-bib-0018] Kostareva, A. , Kiselev, A. , Gudkova, A. , Frishman, G. , Ruepp, A. , Frishman, D. , Smolina, N. , Tarnovskaya, S. , Nilsson, D. , Zlotina, A. , Khodyuchenko, T. , Vershinina, T. , Pervunina, T. , Klyushina, A. , Kozlenok, A. , Sjoberg, G. , Golovljova, I. , Sejersen, T. , & Shlyakhto, E. (2016). Genetic Spectrum of idiopathic restrictive cardiomyopathy uncovered by next‐generation sequencing. PLoS One, 11, e0163362. 27662471 10.1371/journal.pone.0163362 PMC5035084

[dgd12909-bib-0019] Lee, T. M. , Hsu, D. T. , Kantor, P. , Towbin, J. A. , Ware, S. M. , Colan, S. D. , Chung, W. K. , Jefferies, J. L. , Rossano, J. W. , Castleberry, C. D. , Addonizio, L. J. , Lal, A. K. , Lamour, J. M. , Miller, E. M. , Thrush, P. T. , Czachor, J. D. , Razoky, H. , Hill, A. , & Lipshultz, S. E. (2017). Pediatric cardiomyopathies. Circulation Research, 121, 855–873. 28912187 10.1161/CIRCRESAHA.116.309386 PMC5657298

[dgd12909-bib-0020] Li, Y. , Charles, P. Y. , Nan, C. , Pinto, J. R. , Wang, Y. , Liang, J. , Wu, G. , Tian, J. , Feng, H. Z. , Potter, J. D. , Jin, J. P. , & Huang, X. (2010). Correcting diastolic dysfunction by Ca^2+^ desensitizing troponin in a transgenic mouse model of restrictive cardiomyopathy. Journal of Molecular and Cellular Cardiology, 49, 402–411. 20580639 10.1016/j.yjmcc.2010.04.017 PMC5394742

[dgd12909-bib-0021] Maron, B. J. , Towbin, J. A. , Thiene, G. , Antzelevitch, C. , Corrado, D. , Arnett, D. , Moss, A. J. , Seidman, C. E. , & Young, J. B. (2006). Contemporary definitions and classification of the cardiomyopathies: An American Heart Association scientific statement from the council on clinical cardiology, heart failure and transplantation committee; quality of care and outcomes research and functional genomics and translational biology interdisciplinary working groups; and council on epidemiology and prevention. Circulation, 113, 1807–1816. 16567565 10.1161/CIRCULATIONAHA.106.174287

[dgd12909-bib-0022] Martin, T. G. , Myers, V. D. , Dubey, P. , Dubey, S. , Perez, E. , Moravec, C. S. , Willis, M. S. , Feldman, A. M. , & Kirk, J. A. (2021). Cardiomyocyte contractile impairment in heart failure results from reduced BAG3‐mediated sarcomeric protein turnover. Nature Communications, 12, 2942. 10.1038/s41467-021-23272-z PMC8134551 34011988

[dgd12909-bib-0023] Matsa, E. , Burridge, P. W. , & Wu, J. C. (2014). Human stem cells for modeling heart disease and for drug discovery. Science Translational Medicine, 6, 239ps236. 10.1126/scitranslmed.3008921 PMC4215696 24898747

[dgd12909-bib-0024] Mearini, G. , Stimpel, D. , Geertz, B. , Weinberger, F. , Krämer, E. , Schlossarek, S. , Mourot‐Filiatre, J. , Stoehr, A. , Dutsch, A. , Wijnker, P. J. M. , Braren, I. , Katus, H. A. , Müller, O. J. , Voit, T. , Eschenhagen, T. , & Carrier, L. (2014). Mybpc3 gene therapy for neonatal cardiomyopathy enables long‐term disease prevention in mice. Nature Communications, 5, 5515. 10.1038/ncomms6515 25463264

[dgd12909-bib-0025] Miki, K. , Deguchi, K. , Nakanishi‐Koakutsu, M. , Lucena‐Cacace, A. , Kondo, S. , Fujiwara, Y. , Hatani, T. , Sasaki, M. , Naka, Y. , Okubo, C. , Narita, M. , Takei, I. , Napier, S. C. , Sugo, T. , Imaichi, S. , Monjo, T. , Ando, T. , Tamura, N. , Imahashi, K. , … Yoshida, Y. (2021). ERRgamma enhances cardiac maturation with T‐tubule formation in human iPSC‐derived cardiomyocytes. Nature Communications, 12, 3596. 10.1038/s41467-021-23816-3 PMC8217550 34155205

[dgd12909-bib-0026] Miki, K. , Endo, K. , Takahashi, S. , Funakoshi, S. , Takei, I. , Katayama, S. , Toyoda, T. , Kotaka, M. , Takaki, T. , Umeda, M. , Okubo, C. , Nishikawa, M. , Oishi, A. , Narita, M. , Miyashita, I. , Asano, K. , Hayashi, K. , Osafune, K. , Yamanaka, S. , … Yoshida, Y. (2015). Efficient detection and purification of cell populations using synthetic MicroRNA switches. Cell Stem Cell, 16, 699–711. 26004781 10.1016/j.stem.2015.04.005

[dgd12909-bib-0027] Mogensen, J. , Kubo, T. , Duque, M. , Uribe, W. , Shaw, A. , Murphy, R. , Gimeno, J. R. , Elliott, P. , & McKenna, W. J. (2003). Idiopathic restrictive cardiomyopathy is part of the clinical expression of cardiac troponin I mutations. The Journal of Clinical Investigation, 111, 209–216. 12531876 10.1172/JCI16336 PMC151864

[dgd12909-bib-0028] Myers, V. D. , Gerhard, G. S. , McNamara, D. M. , Tomar, D. , Madesh, M. , Kaniper, S. , Ramsey, F. V. , Fisher, S. G. , Ingersoll, R. G. , Kasch‐Semenza, L. , Wang, J. F. , Hanley‐Yanez, K. , Lemster, B. , Schwisow, J. A. , Ambardekar, A. V. , Degann, S. H. , Bristow, M. R. , Sheppard, R. , Alexis, J. D. , … Feldman, A. M. (2018). Association of Variants in BAG3 with cardiomyopathy outcomes in African American individuals. JAMA Cardiology, 3, 929–938. 30140897 10.1001/jamacardio.2018.2541 PMC6233818

[dgd12909-bib-0029] Nguyen, S. N. , Chung, M. M. , Vinogradsky, A. V. , Richmond, M. E. , Zuckerman, W. A. , Goldstone, A. B. , & Bacha, E. A. (2023). Long‐term outcomes of surgery for obstructive hypertrophic cardiomyopathy in a pediatric cohort. JTCVS Open, 15, 726–738. 10.1016/j.xjon.2023.09.032 PMC10775098 38204697

[dgd12909-bib-0030] Pioner, J. M. , Santini, L. , Palandri, C. , Langione, M. , Grandinetti, B. , Querceto, S. , Martella, D. , Mazzantini, C. , Scellini, B. , Giammarino, L. , Lupi, F. , Mazzarotto, F. , Gowran, A. , Rovina, D. , Santoro, R. , Pompilio, G. , Tesi, C. , Parmeggiani, C. , Regnier, M. , … Coppini, R. (2022). Calcium handling maturation and adaptation to increased substrate stiffness in human iPSC‐derived cardiomyocytes: The impact of full‐length dystrophin deficiency. Frontiers in Physiology, 13, 1030920. 36419836 10.3389/fphys.2022.1030920 PMC9676373

[dgd12909-bib-0031] Rapezzi, C. , Aimo, A. , Barison, A. , Emdin, M. , Porcari, A. , Linhart, A. , Keren, A. , Merlo, M. , & Sinagra, G. (2022). Restrictive cardiomyopathy: Definition and diagnosis. European Heart Journal, 43, 4679–4693. 36269634 10.1093/eurheartj/ehac543 PMC9712030

[dgd12909-bib-0032] Rindler, T. N. , Hinton, R. B. , Salomonis, N. , & Ware, S. M. (2017). Molecular characterization of pediatric restrictive cardiomyopathy from integrative genomics. Scientific Reports, 7, 39276. 28098235 10.1038/srep39276 PMC5241776

[dgd12909-bib-0033] Ronaldson‐Bouchard, K. , Ma, S. P. , Yeager, K. , Chen, T. , Song, L. J. , Sirabella, D. , Morikawa, K. , Teles, D. , Yazawa, M. , & Vunjak‐Novakovic, G. (2018). Advanced maturation of human cardiac tissue grown from pluripotent stem cells. Nature, 556, 239–243. 29618819 10.1038/s41586-018-0016-3 PMC5895513

[dgd12909-bib-0034] Russo, L. M. , & Webber, S. A. (2005). Idiopathic restrictive cardiomyopathy in children. Heart, 91, 1199–1202. 16103558 10.1136/hrt.2004.043869 PMC1769097

[dgd12909-bib-0035] Sasse, S. B. J. N. , & Kyprianou, P. (1993). <Circ res 1993 cTnI‐ssTnI fetal neonatal heart.Pdf>. Circ Res.

[dgd12909-bib-0036] Sheng, J. J. , & Jin, J. P. (2016). TNNI1, TNNI2 and TNNI3: Evolution, regulation, and protein structure‐function relationships. Gene, 576, 385–394. 26526134 10.1016/j.gene.2015.10.052 PMC5798203

[dgd12909-bib-0037] Tachampa, K. , Kobayashi, T. , Wang, H. , Martin, A. F. , Biesiadecki, B. J. , Solaro, R. J. , & de Tombe, P. P. (2008). Increased cross‐bridge cycling kinetics after exchange of C‐terminal truncated troponin I in skinned rat cardiac muscle. The Journal of Biological Chemistry, 283, 15114–15121. 18378675 10.1074/jbc.M801636200 PMC2397483

[dgd12909-bib-0038] Takita, K. A. T. , Sasaki, Y. , Higuchi, T. , & Kobayashi, K. (2003). High‐accuracy image registration based on phase‐only correlation. IEICE Transactions on Fundamentals, E86‐A, 1925–1934.

[dgd12909-bib-0039] Towbin, J. A. , Lowe, A. M. , Colan, S. D. , Sleeper, L. A. , Orav, E. J. , Clunie, S. , Messere, J. , Cox, G. F. , Lurie, P. R. , Hsu, D. , Canter, C. , Wilkinson, J. D. , & Lipshultz, S. E. (2006). Incidence, causes, and outcomes of dilated cardiomyopathy in children. JAMA, 296, 1867–1876. 17047217 10.1001/jama.296.15.1867

[dgd12909-bib-0040] Tripathi, S. , Schultz, I. , Becker, E. , Montag, J. , Borchert, B. , Francino, A. , Navarro‐Lopez, F. , Perrot, A. , Özcelik, C. , Osterziel, K. J. , McKenna, W. J. , Brenner, B. , & Kraft, T. (2011). Unequal allelic expression of wild‐type and mutated beta‐myosin in familial hypertrophic cardiomyopathy. Basic Research in Cardiology, 106, 1041–1055. 21769673 10.1007/s00395-011-0205-9 PMC3228959

[dgd12909-bib-0041] Wang, B. Z. , Nash, T. R. , Zhang, X. , Rao, J. , Abriola, L. , Kim, Y. , Zakharov, S. , Kim, M. , Luo, L. J. , Morsink, M. , Liu, B. , Lock, R. I. , Fleischer, S. , Tamargo, M. A. , Bohnen, M. , Welch, C. L. , Chung, W. K. , Marx, S. O. , Surovtseva, Y. V. , … Fine, B. M. (2023). Engineered cardiac tissue model of restrictive cardiomyopathy for drug discovery. Cell Reports Medicine, 4, 100976. 36921598 10.1016/j.xcrm.2023.100976 PMC10040415

[dgd12909-bib-0042] Wang, K. , Schriver, B. J. , Aschar‐Sobbi, R. , Yi, A. Y. , Feric, N. T. , & Graziano, M. P. (2023). Human engineered cardiac tissue model of hypertrophic cardiomyopathy recapitulates key hallmarks of the disease and the effect of chronic mavacamten treatment. Frontiers in Bioengineering and Biotechnology, 11, 1227184. 37771571 10.3389/fbioe.2023.1227184 PMC10523579

[dgd12909-bib-0043] Webber, S. A. , Lipshultz, S. E. , Sleeper, L. A. , Lu, M. , Wilkinson, J. D. , Addonizio, L. J. , Canter, C. E. , Colan, S. D. , Everitt, M. D. , Jefferies, J. L. , Kantor, P. F. , Lamour, J. M. , Margossian, R. , Pahl, E. , Rusconi, P. G. , Towbin, J. A. , & Pediatric Cardiomyopathy Registry Investigators . (2012). Outcomes of restrictive cardiomyopathy in childhood and the influence of phenotype: A report from the pediatric cardiomyopathy registry. Circulation, 126, 1237–1244. 22843787 10.1161/CIRCULATIONAHA.112.104638

[dgd12909-bib-0044] Wen, Y. , Xu, Y. , Wang, Y. , Pinto, J. R. , Potter, J. D. , & Kerrick, W. G. (2009). Functional effects of a restrictive‐cardiomyopathy‐linked cardiac troponin I mutation (R145W) in transgenic mice. Journal of Molecular Biology, 392, 1158–1167. 19651143 10.1016/j.jmb.2009.07.080 PMC2774805

[dgd12909-bib-0045] Yang, X. , Pabon, L. , & Murry, C. E. (2014). Engineering adolescence: Maturation of human pluripotent stem cell‐derived cardiomyocytes. Circulation Research, 114, 511–523. 24481842 10.1161/CIRCRESAHA.114.300558 PMC3955370

[dgd12909-bib-0046] Yazawa, M. , Hsueh, B. , Jia, X. , Pasca, A. M. , Bernstein, J. A. , Hallmayer, J. , & Dolmetsch, R. E. (2011). Using induced pluripotent stem cells to investigate cardiac phenotypes in Timothy syndrome. Nature, 471, 230–234. 21307850 10.1038/nature09855 PMC3077925

[dgd12909-bib-0047] Yumoto, F. , Lu, Q. W. , Morimoto, S. , Tanaka, H. , Kono, N. , Nagata, K. , Ojima, T. , Takahashi‐Yanaga, F. , Miwa, Y. , Sasaguri, T. , Nishita, K. , Tanokura, M. , & Ohtsuki, I. (2005). Drastic Ca^2+^ sensitization of myofilament associated with a small structural change in troponin I in inherited restrictive cardiomyopathy. Biochemical and Biophysical Research Communications, 338, 1519–1526. 16288990 10.1016/j.bbrc.2005.10.116

[dgd12909-bib-0048] Zhao, Y. , Rafatian, N. , Feric, N. T. , Cox, B. J. , Aschar‐Sobbi, R. , Wang, E. Y. , Aggarwal, P. , Zhang, B. , Conant, G. , Ronaldson‐Bouchard, K. , Pahnke, A. , Protze, S. , Lee, J. H. , Huyer, L. D. , Jekic, D. , Wickeler, A. , Naguib, H. E. , Keller, G. M. , Vunjak‐Novakovic, G. , … Radisic, M. (2019). A platform for generation of chamber‐specific cardiac tissues and disease modeling. Cell, 176, 913–927.e18. 30686581 10.1016/j.cell.2018.11.042 PMC6456036

