# Supplementary material for: Gene correction and overexpression of TNNI3 improve impaired relaxation in engineered heart tissue model of pediatric restrictive cardiomyopathy
Source: Dev Growth Differ. 2024 Jan 9;66(2):119–32. doi: 10.1111/dgd.12909 (PMC11457505; doi:10.1111/dgd.12909)
Supplement: Supplementary file 1 — Figure S1. Generation of R170W‐iPSCs and Isogenic‐iPSCs. (a) R170W‐iPSC flow cytometry analysis of the pluripotency markers OCT3/4, SOX2, SSEA4, and TRA‐1‐60. Almost all cells were positive for these markers. (b) The karyotype of R170W‐iPSCs showed a normal male pattern. (c) Direct Sanger sequencing analysis of the R170W‐iPSCs using genomic DNA obtained from the cells. (d) Isogenic‐iPSC flow cytometry analysis of the pluripotency markers OCT3/4, SOX2, SSEA4, and TRA‐1‐60. Almost all cells were positive for these markers. (e) The karyotype of Isogenic‐iPSCs showed a normal male pattern. (f) Direct Sanger sequencing analysis of the Isogenic‐iPSCs using genomic DNA obtained from the cells. Figure S2. Generation of R170W‐TNNI3‐iPSCs. (a) Schematic diagram of transfection of R170W‐iPSCs using the TNNI3 gene vector. (b) Troponin T‐positive ratio in flow cytometric analysis of R170W‐iPSC‐TNNI3‐CMs (n = 5). Data are presented as the mean ± SEM. (c) Results of droplet digital PCR analysis using cDNA samples obtained from R170W‐iPSC‐CMs and R170W‐iPSC‐TNNI3‐CMs. (d) Representative western blot images of R170W‐EHT and R170W‐TNNI3‐EHT lysates 14 days after EHT generation using the indicated antibodies. The molecular weight ladder is shown on the left. Table S1. Sequence information. [file DGD-66-119-s003.pdf]

S Figure 1

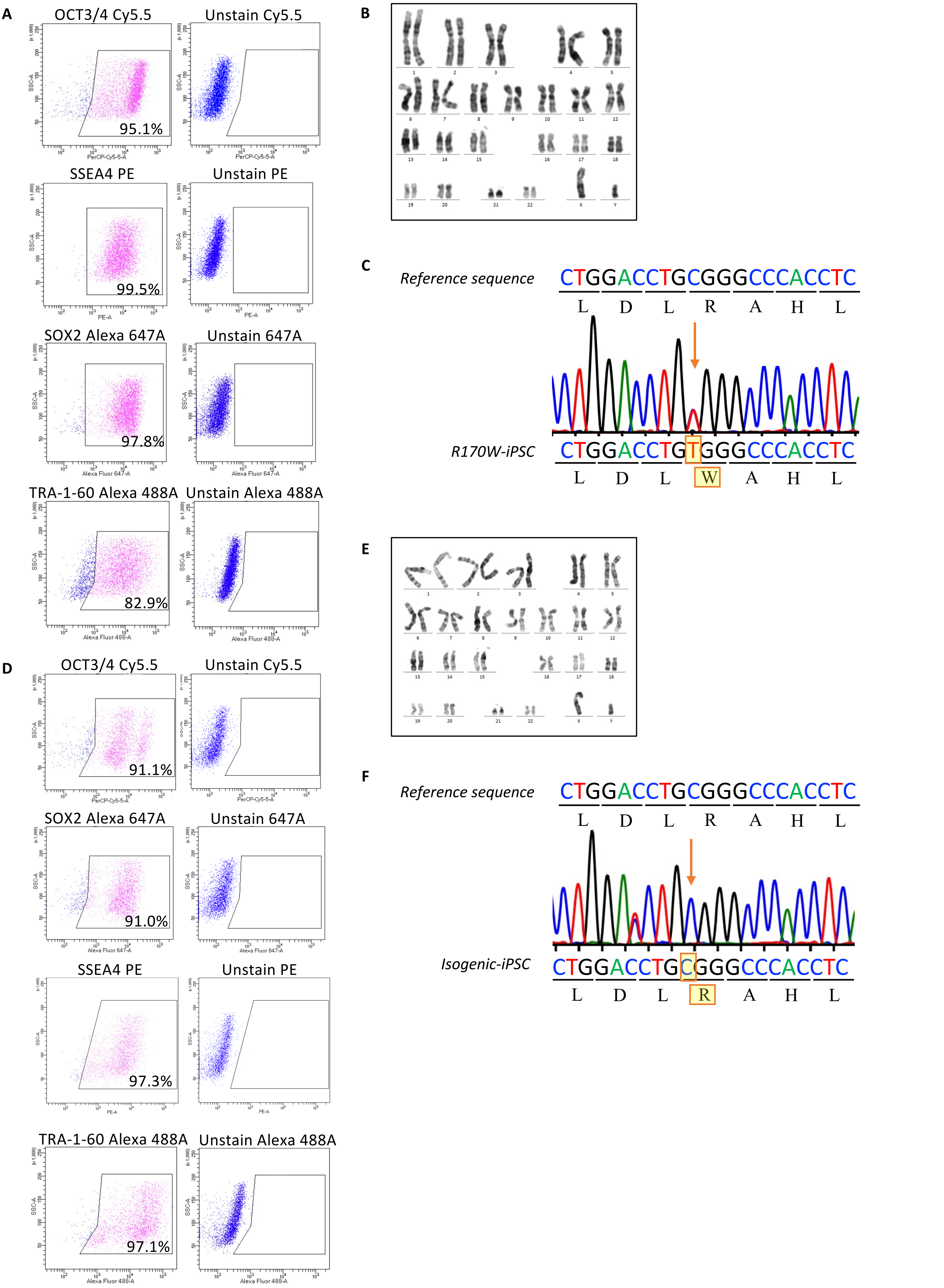
A
OCT3/4 Cy5.5
Unstain Cy5.5
95.1%
SSEA4 PE
Unstain PE
99.5%
SOX2 Alexa 647A
Unstain 647A
97.8%
TRA-1-60 Alexa 488A
Unstain Alexa 488A
82.9%
B
C
Reference sequence
CTGGACCTGCGGGCCCACCTC
L D L R A H L
R170W-iPSC
CTGGACCTGTGGGCCCACCTC
L D L W A H L
D
OCT3/4 Cy5.5
Unstain Cy5.5
91.1%
SOX2 Alexa 647A
Unstain 647A
91.0%
SSEA4 PE
Unstain PE
97.3%
TRA-1-60 Alexa 488A
Unstain Alexa 488A
97.1%
E
F
Reference sequence
CTGGACCTGCGGGCCCACCTC
L D L R A H L
Isogenic-iPSC
CTGGACCTGCGGGCCCACCTC
L D L R A H L

S Figure 2

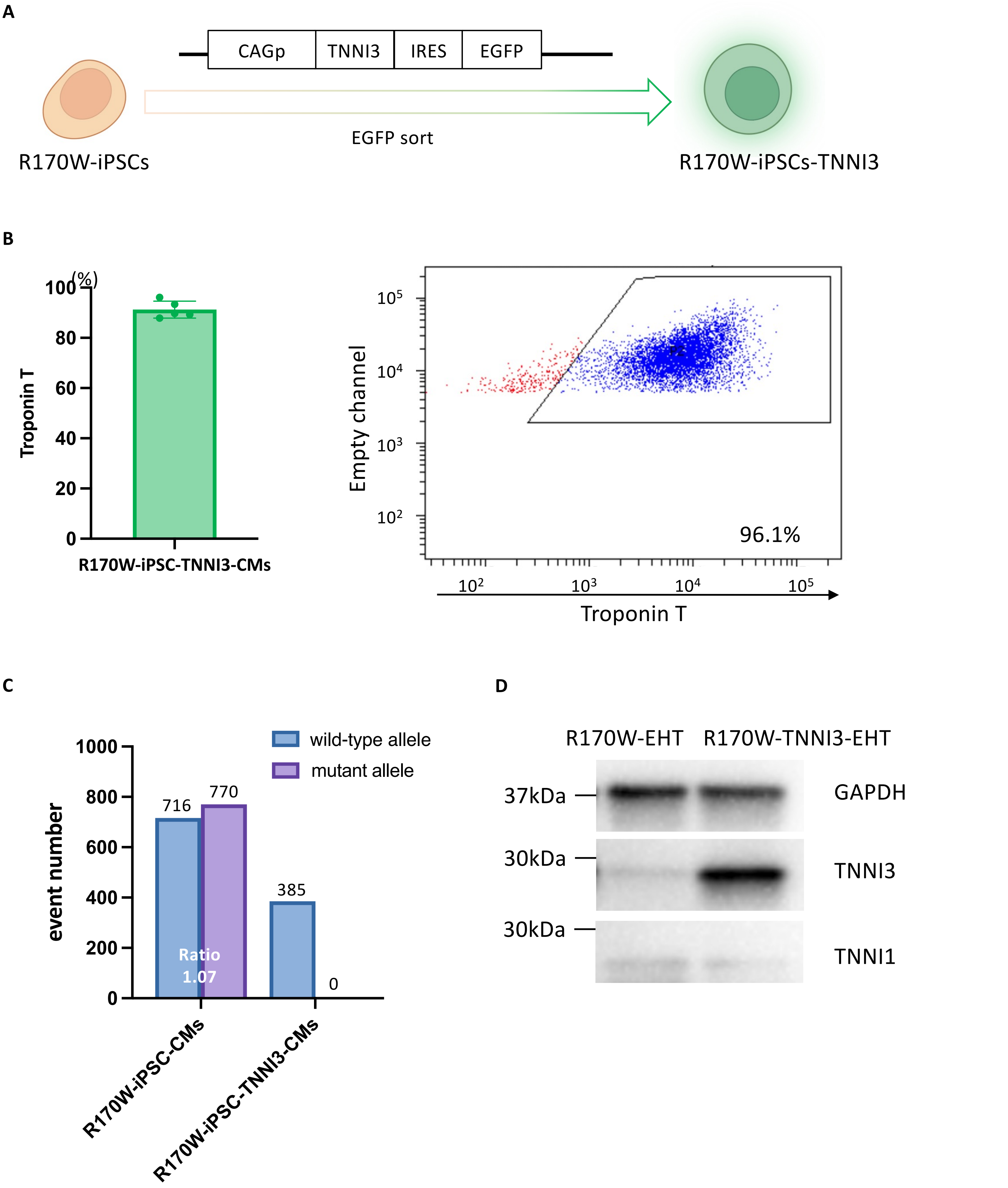
A
CAGp
TNNI3
IRES
EGFP
R170W-iPSCs
EGFP sort
R170W-iPSCs-TNNI3
B
(%)
100
80
60
40
20
0
Troponin T
R170W-iPSC-TNNI3-CMs
Empty channel
10^5
10^4
10^3
10^2
96.1%
Troponin T
C
1000
800
600
400
200
0
event number
wild-type allele
mutant allele
716
770
385
0
Ratio
1.07
R170W-iPSC-CMs
R170W-iPSC-TNNI3-CMs
D
R170W-EHT
R170W-TNNI3-EHT
37kDa
30kDa
30kDa
GAPDH
TNNI3
TNNI1

S Table

	name	sequence	
CRISPR/Cas9 Correction	37/rev gRNA	CCTGCTTGAGGTGGGCCCACAGG	
	42/fw gRNA	TAAGGAGTCCCTGGACCTGTGGG	
	ssODN	GATCTCTGCAGATGCCATGATGCAGGCGCTGCTGGGGG CCCGGGCTAAGGAGTCCCTGGAtCTGcGGGCCCACCTCA AGCAGGTGAAGAAGGAGGACACCGAGAAGgtgagtgtgg gctaaggcc	Blue: Silent mutation site<br>Red: Correction site
Sequence analysis	TNNI3_6149F	GCATGTAGCTGGACCAGGTT	
	TNNI3_6729R2	ACTTCCTGTAGCCCTAATGCAC	
	TNNI3_6266F seq	ACTGGTAAGGCCTCGGTACT	
ddPCR TNNI3	TNNI3_R170W_F	TCTCTGCAGATGCCATGATG	
	TNNI3_R170W_R	TGTTCTTGCGCCAGTCTC	
	TNNI3_Wt probe (FAM)	TGGACCTGCGGG	
	TNNI3_Mut probe (HEX)	CCTGTGGGCC	
